# The natural history of greater trochanteric pain syndrome: an 11-year follow-up study

**DOI:** 10.1186/s12891-021-04935-w

**Published:** 2021-12-20

**Authors:** Luke Bicket, Julie Cooke, Isaac Knott, Angie Fearon

**Affiliations:** 1 Faculty of Health, University of Canberra, 11 Kirinari St, Bruce, ACT 2617 Australia; 2 Research Institute for Sport and Exercise, University of Canberra, 11 Kirinari St, Bruce, ACT 2617 Australia; 3 Trauma and Orthopaedic Research Centre at the Canberra Hospital, Garren, ACT 2606 Australia; 4 Canberra Health Services, 20 Guraguma St, Bruce, 2617 Australia

**Keywords:** Greater trochanteric pain syndrome, GTPS, hip osteoarthritis, Hip OA, hip pain, bursitis, Gluteal tendinopathy, Trochanteric bursitis, follow-up, natural history

## Abstract

**Background:**

Greater trochanteric pain syndrome (GTPS) is a musculoskeletal condition which can cause disability and reduce quality of life. However, limited evidence is available on the long-term outcomes of people with GTPS. Our aims were to determine the long-term prevalence of GTPS; to calculate the proportion of people with GTPS who had developed hip osteoarthritis (OA); and to determine the level of function and quality of life, 11-years after initial GTPS diagnosis.

**Methods:**

A prospective 11-year natural history study. Two groups [GTPS group (*n* = 24), asymptomatic control (ASC) group (*n* = 20)] were evaluated at baseline, 12-months and 11-years. At 11-years all participants completed the modified Harris Hip Score (mHHS), Oswestry Disability Index (ODI) and Assessment of Quality-of-Life questionnaire. At 11-year follow-up 20/24 GTPS and 19/20 ASC participants were clinically assessed for GTPS and hip OA, completed the 10 metre-walk-test, timed up and go, and hip abduction and external rotation strength testing.

**Results:**

At 11-year follow-up 45.0% of GTPS participants had GTPS compared to 5.3% of ASC participants (*p* = 0.008), OR [95% CI]: 10.19 [1.95, 104.3], and 35.0% of GTPS participants were clinically diagnosed with hip OA compared to none of the ASC participants (*p* = 0.002), OR [95% CI]: 21.6, [2.3, 2898.0]. GTPS participants reported more pain and disability than ASC participants via the ODI, mean difference [95% CI]: 6.1 [0.7, 11.6] but not the modified Harris Hip Score, mean difference [95% CI]: -3.3 [-10.3, 3.7]. Both groups had similar levels of quality of life and measures of function.

**Conclusions:**

GTPS is a chronic condition: people with GTPS at baseline had twice the odds of being clinically diagnosed with GTPS or hip OA than the control group at 11-years. Further, there appears to be a temporal relationship between GTPS and the development of hip OA. This finding highlights the need to identify effective treatments that address the underlying impairments associated with GTPS. Pain and function results varied depending on the assessment tools used. Between group differences in quality of life seen at baseline are not found at the 11-year follow-up. The small sample size means the results must be considered with caution.

**Level of Evidence:**

Level II Natural history Study.

**Supplementary Information:**

The online version contains supplementary material available at 10.1186/s12891-021-04935-w.

## Background

Greater trochanteric pain syndrome (GTPS) causes pain on the lateral side of the hip with subsequent dysfunction which negatively impacts quality of life and reduces the ability to remain in full-time work, compared to healthy age matched controls [1]. The underlying pathology is thought to primarily be due to gluteus medius and gluteus minimus tendinopathy [2, 3]. GTPS is more common than Achilles tendinopathy, with a prevalence of 4.22/1000 person-years, and an incidence rate of 3.29/1000 person-years [4], yet there is little long-term data about this condition.

The single prognostic study on GTPS (*n* = 164) reported at least 36% of people at 1-year and 29% of people at 5-years post diagnosis still had GTPS, with 24% self-reporting concurrent hip osteoarthritis (OA) [5]. While informative, this primary care, GP based study was limited by the low follow-up rate (54%), the retrospective design, lack of a clinical interview or examination to confirm the diagnosis and the absence of imaging findings at any point in the study. To our knowledge there is no long-term study that reports on dysfunction, quality of life or function in people with GTPS.

GTPS has been linked to end-stage hip OA [6–8]. The incidence of gluteal tendon tears, likely severe GTPS [1], identified at hip arthroplasty ranges from 1.6% [6] to 20% [7], suggesting a mild to moderate association between GTPS and end-stage hip OA. However, there are no longitudinal studies to indicate if the conditions arise concurrently or sequentially. A clearer understanding of the relationship between GTPS and hip OA is important for further research into the treatment of GTPS and consequently patient management.

The dysfunction and quality of life of people with GTPS, and those with gluteal tendon tears has been shown to be poor compared to an aged and sex matched asymptomatic control group, and similar to people with end stage hip OA [1, 9]. To our knowledge, there are no longitudinal studies that have examined if these finding persist over time.

In summary, there is a lack of evidence surrounding the long-term outcomes of GTPS and potential associations with ongoing GTPS and hip OA. Therefore, our research questions were:

1. What is the GTPS status of a GTPS cohort and an asymptomatic control cohort at 11-years follow-up?
2. What is the hip OA status of a GTPS cohort and an asymptomatic control cohort at 11-years follow-up?
3. What are the comparative (disability, quality of life and clinical function) outcomes of people with GTPS and an asymptomatic control cohort at one and 11-years following initial assessment for GTPS?

## Methods

### Study design and setting

This prospective cohort study was completed in a university setting at three time points: baseline (2008), 12-months (2009), and 11-years (2019). Between baseline and 12-month follow-up participants received intermittent (approximately second monthly) correspondence with the aim of reducing attrition. This study was approved by university human research ethics committees (HREC: 20181528), all participants provided informed consent. Previous consent had been obtained from the participants at the baseline assessment for later follow-up, all participants reconsented for the 2019 follow-up assessment.

### Participants

A total of 85 participants were originally recruited from the local community via professional networks and word-of-mouth between March 2008 and November 2009. The initial sample included three mutually exclusive groups, a GTPS group (*n* = 42, 11 of whom underwent gluteal reconstruction surgery in the following 12 months), a hip OA group (*n* = 20, all who underwent total hip arthroplasty (THA) in the following 12 months), and an asymptomatic control group (ASC) (*n* = 23) of similar age and sex to the GTPS participants. This 11-year follow-up study reviews a subset of the original cohort, see exclusion criteria for the 11-year follow-up study.

Inclusion criteria for this study (11-year follow-up) was having previously been involved in the GTPS study of 11-years ago [1, 10]. Relevant inclusion criteria for the baseline study were being over 18-years of age, able to communicate in English and a) GTPS group: having a clinical diagnosis of GTPS (minimum three-month history of lateral hip pain, pain on palpation of the greater trochanter, and pain with either lying on the hip during weight bearing, or sitting), or b) asymptomatic control group (ASC): having no history of lower limb injury or disease. Exclusion criteria at baseline for both groups were evidence of intra-articular hip joint pathology (clinically: reporting groin pain, groin pain with any hip examination test (e.g. FADDIR/FABER/internal rotation) and on imaging (x-ray or MRI)), systemic inflammatory disease, a history of hip or spinal surgery, for the GTPS group a cortisone injection into the lateral hip within the last 3 months and for the ASC group any history of hip pain [1, 10].

Exclusion criteria for the 11-year follow-up study were participants from the original cohort who underwent surgery in the 12 months following baseline assessment, (gluteal tendon reconstructive surgery (*n* = 11) or THA (*n* = 20)).

**Fig. 1:**
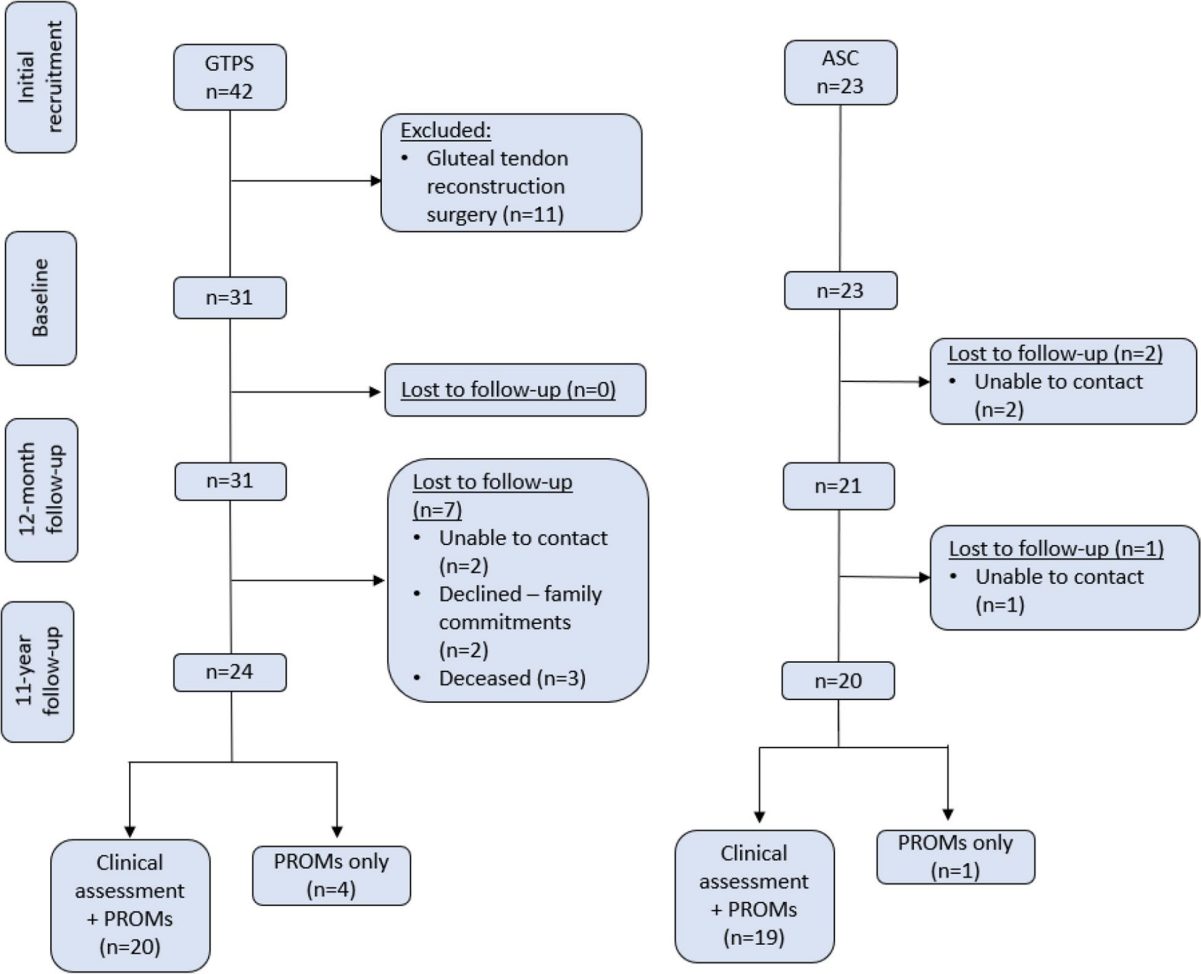
Flow of participants in 11-year follow-up study

We attempted to contact all remaining participants (*n* = 54), regardless of their symptomatic status, to invite to take part in this 11-year follow-up study. For clarity we have continued to use the descriptors GTPS and ASC for the groups regardless of their current diagnosis. Following email, postal mail, phone calls and checking the electoral roll we were unable to contact five previous participants (two GTPS and three ASC), we were informed that three GTPS participants had passed away, and two GTPS participants declined to be involved, leaving GTPS *n* = 24, ASC *n* = 20 (Fig. 1).

All the 11-year follow-up assessments were performed by a successfully blinded assessor (LB), a final year physiotherapy student, trained and supervised in the assessment techniques by the senior author who has 20 years of clinical experience in diagnosing hip conditions (AF).

### Participant demographic details

Participant age, sex, weight, height, receipt of corticosteroid injections, and employment status were recorded as these have previously been associated with GTPS [1, 8] and may have been co-variants during data analysis. At baseline, the most affected leg was chosen as the index leg for the GTPS group. All data presented in this study including patient reported and clinical examination results relate to the index leg. For the ASC group the “index leg” was randomly assigned as baseline analysis found no between leg hip abduction strength difference (ICC (C, 1) (95% CI) = 0.86 (0.70 to 0.94)). Data from the same leg is reported for each data collection point.

### Clinical diagnosis

To determine the clinical diagnosis of GTPS at 11-years follow-up we used the same criteria as at baseline: a minimum three-month history of lateral hip pain, pain on palpation of the greater trochanter, and pain with either lying on the hip, during weight bearing, or sitting [1].

To determine the clinical diagnosis of hip OA we used Altman (1991) criteria: history of hip pain and internal rotation <15° and flexion <115°, or a history of hip pain, pain on internal rotation, morning stiffness ≤60 minutes, and age >50 years [11]. Where measures were close to these cut off (<15° of internal rotation in 90° flexion, or <115° flexion in supine) a goniometer was used to measure the range*.* Alternatively, a history of total hip arthroplasty for hip OA on the affected side was considered a diagnosis of hip OA [12]. Following the publication of a recent systematic review on clinically diagnosing hip OA [13], we undertook a post-hoc determination of hip OA diagnosis based on our existing data (Additional file 1). Where a person presented at 11-year follow-up with groin or lateral hip pain but did not fit the Altman criteria, they were categorised as having “other source of hip pain”.

### Disability and quality of life

In the absence of a condition specific measure for this population at baseline, measures with face validity were used. To enable a longitudinal comparison, we chose to repeat those measures at the 11-year follow-up.

As per the baseline [1, 10], we assessed:

- quality of life with the Assessment of Quality of Life questionnaire (AQoL 4-D [14, 15])
- disability via the modified Harris Hip Score (mHHS) [16] and the Oswestry Disability Index (ODI) [17]. For the ODI, participants were asked to respond in relation to their leg pain, rather than any existing back pain.
- the number of co-morbidities via the Functional Co-morbidities Index (FCI) [18]

### Functional outcomes

As per the baseline, four reliable and valid clinical tests were undertaken by LB. As noted above LB was trained and supervised by AF.

- Hip abduction and external rotation strength, normalised to mass (kgf/m^BMavg^) [19]
- Gait speed via the 10-meter walk test (10mwt) (m/s) [20]
- Timed up and go (TUG) (s) [21]

Maximum isometric hip abduction (in supine) and external rotation strength (in prone) were assessed using the same fixed calibrated hand-held dynamometer, *(Chatillon, MSC FL, USA)* as used at baseline assessment" [10]. The 10mwt was completed four times with participants instructed to walk at a “fast and safe pace” and to start on “ready, set, go!”. The TUG was performed in the standard manner, however the instructions were inadvertently varied from the original study [10]. At each time point, the average of these measures is reported.

### Sample size calculation

In this natural history study, we attempted to contact all eligible previous participants from the baseline exploratory study [1, 10] via email, letters, phone calls and a search on the Australian electoral role, thus no sample size calculation was undertaken.

### Statistical analysis

The baseline and 12-month data from the two surgical groups were not included in the 11-year follow-up analysis. Data were visually assessed for normality and found to be skewed. Demographic measures are presented using median and interquartile ranges (IQR). For continuous data, between group differences were examined using independent-samples Mann-Whitney U tests. For categorical data (sex, obesity, number of cortisone injections and full-time work status), Chi-square (*X*^2^) analysis was undertaken, except where cell frequency was less than five, when a Fisher Exact test was implemented.

To account for body size effect on hip strength, strength data was standardised to participant body mass via the body mass average index (BMavg) [19].

To answer question 1 and 2, we undertook Fisher Exact evaluations. The post-hoc analysis of the alternative method to diagnose hip OA did not change the overall outcome (Additional file 1), thus we report using the Altman criteria [11]. The Odds Ratio (OR) [95%CI] of having GTPS, or developing hip OA were calculated post hoc using ‘penalized logistic regression (Firth method [22]) as implemented in R package ‘logistf’ [23] , with confidence intervals from the profile likelihood [24].

To answer question 3, we built linear mixed models with group as the fixed effect and age and co-morbidities (FCI) as co-variates at each time point, having found work-status and the number of cortico-steroid injections that a participant reported did not alter outcomes. The results are reported as estimated marginal means (EMM), standard error of the mean (SEM), differences between group means and 95% confidence intervals. SPSS version 22.0 (IBM Corp. Released 2017.Version 25.0. Armonk, NY) was used for all statistical analyses, except for the odds ratio, which was calculated using R (RStudio Team (2020). RStudio: Integrated Development for R. RStudio, PBC, Boston, MA URL http://www.rstudio.com/). Significance level was set at *p* < 0.05.

## Results

### Demographics

We recruited 44 (GTPS = 24, ASC = 20) of the 54 eligible past participants. We clinically assessed 20 (64.5%) and recorded patient reported outcomes for 24 (77.4%) of eligible GTPS participants. We clinically assessed 19 (82.6%) and recorded patient reported outcomes for 20 (86.9%) of eligible ASC participants. A total of five participants could not attend the clinical examination due to living interstate, or carers duties prohibiting their attendance (Fig. 1). Participants were matched for age and sex at baseline and had comparable age and sex at subsequent assessments. Group differences were seen for BMI, but not obesity. GTPS participants had more co-morbidities than the ASC group at baseline and at 12-month follow-up, but not at the 11-year follow-up, Table 1. The GTPS group had more corticosteroid injections and more hip arthroplasties, while full-time work status varied across the years, Table 2.

**Table 1:** Participant Characteristics with Measures of Central Tendencies at Baseline, 12-month and 11-year follow-up for age, BMI and the number of co-morbidities. Between group differences evaluated with between the Median (IQR) via the Independent-samples Mann-Whitney U Test^a^

Characteristic	Baseline				12-month follow-up				11-year follow-up			
	Groups		Difference between groups		Groups		Difference between groups		Groups		Difference between groups	
	GTPS (*n* = 31)	ASC (*n* = 23)	GTPS minus ASC	Mann-Whitney U Test	GTPS (*n* = 31)	ASC (n = 21)	GTPS minus ASC	Mann-Whitney U Test	GTPS (*n* = 24)	ASC (n = 20)	GTPS minus ASC	Mann-Whitney U Test
Age *(yr)*	56.0 (43.0-62.0)	49.0 (43.0-61.0)	7.0	*p* = 0.706	57.0 (44.0-63.0)	58.0 (44.0-62.5)	-1.0	*p* = 0.911	62.0 (52.5-72.0)	63.0 (53.3-72.0)	-1.0	*p* = 0.878
BMI *(kg/m*^*2*^)	27.3 (24.7 to 30.7)	24.2 (22.8 to 27.7)	3.1	***p***=0.035*	27.3 (24.4 to 29.4)	24.1 (22.5 to 26.8)	3.2	***p***=0.031*	25.9 (24.5 to 29.9)	25.4 (22.5 to 29.4)	0.5	*p* = 0.572
Comorbidities *(FCI - number)*	3 (1.0-4.0)	1 (0.0-2.0)	2	***p***=0.006*	2 (1.0 to 4.0)	1 (1.0 to 1.5)	1	***p***=0.007*	2 (1.0-3.8)	2 (1.0-2.0)	0	*p*=0.175

*Note: GTPS *Greater Trochanteric Pain Syndrome, *ASC *Asymptomatic Control Group, *yr *year, *FCI *Functional Co-morbidity Index, *BMI *Body Mass Index, *IQR *Interquartile range, *Statistically significant* (p < 0.05).*

^a^Baseline and 12-month data does not include those excluded from 11-year follow-up, but does include those lost to follow-up

**Table 2:** Participant Characteristics for sex, obesity, number of corticosteroid injections and hip arthroplasty surgery. Number (%), evaluated using X ^2^ or Fisher Exact Test^a^

Characteristic	Baseline			12-month follow-up			11-year follow-up		
	Groups		GTPS vs ASC	Groups		GTPS vs ASC	Groups		GTPS vs ASC
	GTPS (*n* = 31)	ASC (*n* = 23)	*X*^2^/Fischer exact	GTPS (*n* = 31)	ASC (*n* = 21)	*X*^2^/Fischer exact	GTPS (*n* = 24)	ASC (*n* = 20)	*X*^2^/Fischer exact
Female,	28 (90.3)	22 (95.7)	*p*=0.628	28 (90.3)	20 (95.2)	*p*=0.639	22 (91.7)	19 (95.0)	*p*=1.000
Obese *(BMI ≥ 30.0)*,	9 (29.0)	2 (8.7)	*p*=0.092	6 (19.4)	0 (0.0)	*p*=0.070	5 (20.8)	4 (20.0)	*p*=1.000
People who had had a CSI (cumulative number (%))	7 (22.6)	0 (0.0)	***p***=0.015*	14 (45.2)	0 (0.0)	***p***<0.001*	17 (70.8)	1 (5.0)	***p***<0.001*
Full-time work,	13 (41.9)	16 (69.6)	*p*=0.057	10 (32.3)	13 (61.9)	***p***=0.048*	6 (25.0)	7 (35.0)	*p*=0.522
THA on affected leg (Number (%))	0	0		0	0		4 (16.7)	0	

*Note: GTPS* Greater trochanteric pain syndrome; Where cell values were less than 5 Fisher Exact tests was used. *Statistically significant* (p < 0.05).*

*CSI *Corticosteroid injection - numbers carried forwards.

^a^ Baseline and 12-month data does not include those excluded from 11-year follow-up, but does include those lost to follow-up

### Outcomes

**Fig. 2:**
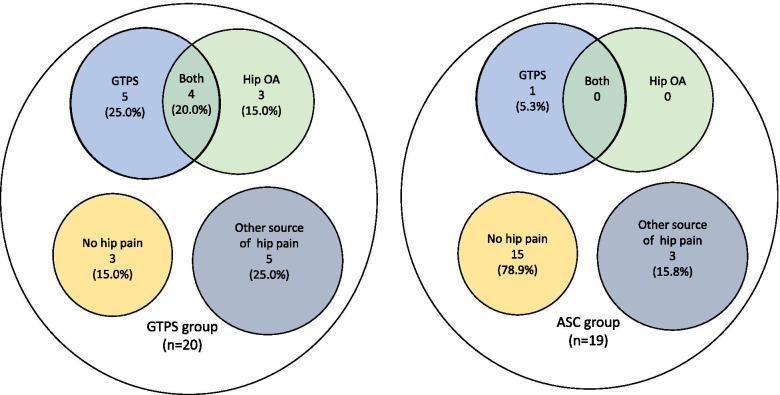
Clinical diagnosis of hip OA based on Altman’s criteria [11] at 11-years of the GTPS group (on the left) and ASC group (on the right). Note, two GTPS participants who were not clinically assessed reported having undergone a total hip arthroplasty. They are not included in this analysis

At 11-year follow-up a larger proportion of GTPS participants had a clinical diagnosis of GTPS than the ASC participants, (Fisher exact, *p* = 0.008), OR [95% CI]: 10.19 [1.95, 104.3]. A large proportion of GTPS participants had gone on to develop hip OA, while none of the ASC had gone on to develop hip OA according to Altman’s criteria (Fisher exact, *p* = 0.002), OR [95%CI] = 21.6 [2.30, 2898.0]. 18.2% of the GTPS had both GTPS and hip OA, while close to 80% of the ASC remained free of hip pain, Fig. 2.

### Patient reported and functional outcomes at 12-months and 11-years

GTPS participants had a lower quality of life than ASC participants at baseline (*p* = 0.004). This difference was not present at 12-months, or at 11-years. GTPS participants reported more disability on the mHHS and the ODI at baseline and 12-months, (*p* < 0.04) and on the ODI (but not the mHHS) at 11-years (*p* = 0.028), Table 2.

There was no group difference for hip abductor or external rotator strength at baseline or 12-month follow-up. The GTPS group had weaker hip abduction than the ASC at 11-years follow-up (*p* = 0.032). The GTPS group walked more slowly at baseline (gait speed, *p* < 0.001) than the ASC, and were slower on the TUG at the 11-year follow-up than the ASC, Table 2.

## Discussion

In this cohort, people with GTPS were more likely to continue to experience hip pain 11-years after baseline assessment when compared to an ASC group. Further, a significantly higher proportion of people with GTPS went on to develop hip OA, than the ASC group. We also found that many of the dysfunctions, quality of life and functional outcome differences seen at baseline between the GTPS and ASC groups had diminished, or no longer existed, at 11-years follow-up (Table 3).

**Table 3:** Patient Reported Outcome Measures, Hip Strength and Gait Parameters Measured Across 11 years. Generalised linear models controlling for age and comorbidities provided estimated marginal means (SE) and 95% ci at each time point

Outcome	Estimated Marginal Means (SE) and 95% CI by Group and Assessment Time Point. Controlling for age and FCI^b^								
	Baseline Age=52.76, FCI=1.96			12-month follow-up Age=54.2, FCI=1.73			11-year follow-up Age=62.5yrs, FCI=2.18		
	GTPS (*n* = 31)	ASC (*n* = 23)	Difference [95% CI]	GTPS (*n* = 31)	ASC (*n* = 21)	Difference [95% CI]	GTPS (*n* = 24)	ASC (*n* = 20)	Difference [95% CI]
AQoL (utility 0-1)	0.78 (0.02) [0.74, 0.82]	0.89 (0.02) [0.83, 0.93]	**-0.10**^a^ **[-0.17, -0.03]**	0.88 (0.02) [0.84, 0.93]	0.85 (0.03) [0.792, 0.90]	0.03 [-0.05, 0.11]	0.84 (0.02) [0.80, 0.88]	0.87 (0.02) [0.82, 0.91]	-0.23 [-0.08, 0.04]
mHHS (0-91)	67.8 (1.9) [64.0, 71.6]	87.2 (2.2) [82.7, 91.7]	**-19.4**^a^ **[-25.6, -13.1]**	77.1 (1.6) [73.9, 80.2]	82.8 (1.9) [78.9, 86.6]	**-5.7**^a^ **[-10.9, -0.47]**	79.2 (2.3) [74.6, 83.8]	82.5 (2.5) [77.5, 87.6]	-3.3 [-10.3, 3.7]
ODI (0-100)	20.2 (1.3) [17.5, 22.9)	3.5 (1.6) [0.3, 6.7)	**16.7**^a^ **[12.3, 21.1)**	12.5 (1.4) [9.6, 15.4)	4.7 (1.8) [1.1, 8.3)	**7.8**^a^ **[3.0, 12.7)**	12.8 (1.8) [9.2, 16.4)	6.6 (2.0) [2.7, 10.6)	**6.1**^a^ **[0.7, 11.6)**
							n = 20	n = 19	
Strength (Hip Abd (N/BM^BMavg^))	36.9 (4.1) [28.9, 44.9]	45.1 (4.8) [35.7, 54.4]	-8.2 (4.3) [-16.6, 0.24]	40.7 (3.9) [33.0, 48.4]	42.5 (4.6) [33.4, 51.6]	-1.8 (4.2) [-10.1, 6.5]	29.1 (6.0) [17.4, 40.8]	39.6 (6.5) [26.8, 52.5]	--10.5 [--21.2, 0.2]
Strength (Hip ER (N/BM^BMavg^))	17.1 (1.5) [14.1, 20.2]	18.8 (1.8) [15.2, 22.3]	--1.7 (1.6) [-4.8, 1.5]	18.7 (1.5) [15.8, 21.6]	19.2 (1.7) [15.8, 22.6]	-0.53 (1.6) [-3.6, 2.6]	18.9 (3.5)^c^ [12.1, 25.6]	21.2 (3.5) [14.2, 28.1]	-2.3 (2.7) [-7.5, 2.9]
TUG (sec)^d^	9.3 (0.40)^d^ [8.5, 10.1]	8.6 (0.43) [7.7, 9.5]	0.7 (0.63) [-0.6, 1.9]	8.8 (0.42) [8.0, 9.7]	8.9 (0.52) [7.8, 9.9]	-0.09 (0.71) [-1.5, 1.3]	6.6 (0.22) [6.2, 7.1]	6.0 (0.23) [5.5, 6.4]	**0.7**^a^ **[0.01, 1.3]**
Gait Speed (m/s)^d^	1.1 (0.39)^d^ [1.0, 1.2]	1.3 (0.04)^c^ [1.3, 1.4]	-0.1^a^ (0.03) [-0.3, 0.0]	1.3 (0.03)^c^ [1.2, 1.3]	1.3 (0.04) [1.2, 1.3]	0.01 [-0.1, 0.1]	1.8 (0.06) [1.6, 1.9]	1.9 (0.06) [1.7, 2.0]	-0.1 [-0.3, 0.0]

^a^ Indicates a statistically significant finding

*AQoL *Higher score indicates higher quality of life

*mHHS *Higher score indicates higher function and less pain

*Strength:* Higher score indicates higher strength

*Gait speed:* Higher score in more desirable

*Time up and go:* lower score is more desirable.

^b^ Baseline and 12-month data does not include those excluded from 11 year follow-up, but does include those lost to follow-up

^c^ One participant requested this examination be ceased due to hip pain. §: Baseline TUG: GTPS *n* = 27, Gait speed: GTPS, *n* = 26, ASC, *n*= 22. 12-month follow-up gait speed: GTPS n = 30.

^d^ Different instructions were inadvertently used at the 11-year follow-up than baseline.

Hip pain is a common cause of pain and disability in older people [25]. Our findings support and extend the findings of Lievense (2005) [5] who, in a retrospective questionnaire based study found 29% of participants continued to report GTPS symptoms, with 24% reporting both GTPS and hip OA diagnosis at five years follow-up. As no clinical assessment was reported for their baseline or follow-up data, it is not clear if the hip OA existed at baseline, or if a higher proportion of their cohort may have had hip OA. In contrast our prospectively collected data showed our participants had no clinical or imaging evidence of intra-articular hip joint pathology at baseline. The increased rate of hip OA in our cohort is likely explained by the longer follow-up period, allowing a longer duration for hip OA to develop.

Our findings support a temporal association between GTPS and hip OA which has not previously been identified, meaning that a clinical diagnosis of GTPS likely results in a higher chance of developing symptomatic hip OA. In 60-64 year old people the estimated prevalence of radiological diagnosis of hip OA ranges from 0.5%-11.5% [26]. Our GTPS participants demonstrated a much higher rate than this. Associations between tendon degeneration and OA have previously been identified at the shoulder [27], between hip OA and obturator internus tendinosis [28], and between the gluteal tendon tears in those undergoing hip arthroplasty surgery [6, 7]. It has been hypothesised that the increased hip adductor moment seen in people with GTPS puts greater load on the gluteal tendons resulting in the persistence of GTPS [29] and similarly, increases the load within the joint [30], possibly contributing to the development of hip OA. Gluteus medius and minimus muscles contribute to stabilising the head of femur within the acetabulum whilst walking [31]. Thus, pathology of this muscular tendinous complex may contribute to, or precede the development of hip OA. We note that our two groups had no difference in strength so other factors such as motor control or somatosensory impairments may also be contributing factors. There is a small amount of evidence suggesting that individuals with GTPS have somatosensory impairments [32, 33], which could contribute to the development of hip OA. Thus, addressing this issue early may be beneficial.

The baseline higher levels of disability and lower quality of life seen in people with GTPS compared to controls were not consistently found at the 11-year follow-up. At the 12-month follow-up, our GTPS cohort reported apparent improvements in quality of life. Further, at this time there appeared to be a smaller between group difference in the disability scores (mHHS and ODI). By 11-years, only the ODI continued to have a (small) between group difference, noting that ODI has been found to measure a different construct than the subsequently developed condition specific VISA-G [34]. The changes after 12-months in the GTPS participants may be due to the Hawthorne effect [35]. The lack of between group difference seen at 11-year years may be due, in part, to the increased rate of hip pain (~20%), in the ASC group which did not exist at baseline, and the increased number of comorbidities in that group. While the participants who were not followed up were not statistically different from those who were (Additional file 2), we still controlled for age and co-morbidities thus reducing the risk of a type two error. The limited sample size may affect these data in relation to function. The only other long-term follow-up paper reports the quality of life and function of those with pain is lower than those without pain [5]. We did not undertake that analysis.

### Limitations

We acknowledge several limitations within this project. Firstly, the 11-year follow-up diagnosis of hip OA was performed in the absence of an x-ray. The diagnosis of hip OA is more accurate when performed in combination with a radiographic examination [11]. However, we repeated the clinical examination using alternative criteria [13], with very similar results. Further, Kim et al (2015) demonstrated a potential inconsistency between clinical and radiographic diagnosis of early OA in the hip [36] meaning that imaging may have biased our results. In addition, we question whether it would be ethical to expose the participants to hip x-rays when it is not difficult to clinically diagnose symptomatic hip OA [13, 37]. We acknowledge that the use of ultra-sound and/or magnetic resonance imaging would have enhanced the diagnosis of GTPS. Secondly, while we had a small sample size, we achieved a high follow-up rate of 77% (GTPS) and 87% (ASC) after 11-years, further, our post-hoc power analysis indicates we had an adequately powered study for determining the risk of hip OA. Nonetheless the small sample size indicates that these results should be considered with caution. Thirdly, the 10mwt and TUG were inadvertently conducted differently at the 11-year follow-up compared to the baseline and 12-month follow-up as participants were asked to walk as fast as they could safely, while at the baseline and 12-month data points, participants were instructed to walk at a self-selected pace when completing these tests. Nevertheless, we can confidently compare between groups at each time point but not within the groups across the different follow-up periods. In addition, we only evaluated the original symptomatic leg, thus clinical tests that relied on both legs may have been compromised. Finally, we did not undertake reliability studies, however LB was trained and supervised by AF – who undertook the original study, and we used outcomes with published high inter-rater reliability.

### Conclusions

This study demonstrates that many people with GTPS continue to experience hip pain (including due to GTPS) and appear to have a higher chance of developing hip OA after 11-years than a comparison group. These findings should be confirmed with a larger study. Clinicians involved in treating people with GTPS should be aware that over time their management strategies may need to change. Future studies should investigate biomechanical and somatosensory impairments and their effects on joint contact forces. Furthermore, investigations exploring effective long-term conservative management strategies are necessary to reduce the burden of GTPS, and the risk of developing hip OA.

## Supplementary Information

**Additional file 1.**

**Additional file 2.**

## Data Availability

The University of Canberra is currently setting up a data repository platform within Mendeley. It is expected that this will be functional within the next six months. Subject to manuscript acceptance, at that point, the data from this study will be uploaded to that platform. Until that time the datasets used and/or analysed during the current study available from the corresponding author on reasonable request.

## Abbreviations

10mwt: 10 metre walk test
ASC: Asymptomatic control
AQoL: Assessment of Quality of Life
FCI: Functional Co-morbidity Index
GTPS: Greater Trochanteric Pain Syndrome
GT: Gluteal Tendon
kgf: kilogram x force
kgf/m^BMavg^: (kilogram x force)/metre^BodyMassAverage^
mHHS: Modified Harris Hip Score
m: metres
OA: Osteoarthritis
ODI: Oswestry Disability Index
TUG: Timed Up and Go
VISA-G: Victorian Institute of Sport – Gluteal
