# Supplementary material for: The natural history of greater trochanteric pain syndrome: an 11-year follow-up study
Source: BMC Musculoskelet Disord. 2021 Dec 20;22:1048. doi: 10.1186/s12891-021-04935-w (PMC8691027; doi:10.1186/s12891-021-04935-w)
Supplement: Supplementary file 1 — Additional file 1. [file 12891_2021_4935_MOESM1_ESM.docx]

## Post-hoc analysis of an alternative Hip OA diagnostic method (1)

In the absence of radiological findings, we chose to undertake an additional clinical method of evaluating the likelihood of someone having hip OA. We included one likelihood ratio from each category of: pain complaints; pain provocation tests; and range of movement (Table 1). Note, we did not specifically examine for some of these clinical tests and have reported those tests we did not exam as negative. Using an estimated hip OA pre-test probability of 10% (2),of those examined, 7 GTPS and 1 ASG were found to have a post-test probability of having hip OA that was greater or equal to 50%, (or a THA, which we accepted as hip OA), according to the likelihood ratios provided by Metcalfe et al (1).

Using the alternative post-hoc clinical diagnosis of hip OA, a larger proportion of GTPS participants (7/20, 35%) had a clinical diagnosis of hip OA compared to ASC participants (1/19, 5%) (Fisher exact, p=0.044). Approximately 80% of those diagnosis with hip OA in GTPS group were diagnosed with OA regardless of the clinical method employed.

|  | **Symptoms** | | | | **Pain Provocation Tests** | | | | **Reduced Range of Motion (Hip)** | | | | | |
| --- | --- | --- | --- | --- | --- | --- | --- | --- | --- | --- | --- | --- | --- | --- |
| **Test** | Pain medial thigh | Pain with stair/slope | Morning stiffness | Limp | Squat | Groin pain with Abd/ Add | Scour | Pain with  hip IR | Restricted 3 planes (if FABER/ FADDIR stiff, or independent ROM) | Restricted 2 planes | Restricted 1 plane | Reduced Add. | Reduced IR | Reduced Abd |
| +LR | 7.8 | 2.1 | 1.5 | 1.5 | 6.1 | 5.7 | 2.4 | 1.4 | 4.4 | 1.5 | 1.3 | 4.2 | 3.2 | 1.6 |
| - LR | 0.89 | 0.47 | 0.22 | 0.35 | 0.79 | 0.71 | 0.51 | 0.31 | NA | NA | NA | 0.25 | 0.43 | 0.26 |

Table 1. Positive and negative likelihood ratios for the diagnosis of Hip OA (1).

+LR: positive likelihood ratio, -LR: negative likelihood ratio, Abd: Abduction, Add: Adduction, IR: Internal rotation, FABER: Flexion abduction external rotation, FADIIR: Flexion adduction internal rotation.

1. Metcalfe D, Perry DC, Claireaux HA, Simel DL, Zogg CK, Costa ML. Does This Patient Have Hip Osteoarthritis?: The Rational Clinical Examination Systematic Review. JAMA. 2019;322(23):2323-33.

2. Kim C, Linsenmeyer KD, Vlad SC, Guermazi A, Clancy MM, Niu J, et al. Prevalence of radiographic and symptomatic hip osteoarthritis in an urban United States community: the Framingham osteoarthritis study. Arthritis Rheumatol. 2014;66(11):3013-7.
