# Supplementary material for: The natural history of greater trochanteric pain syndrome: an 11-year follow-up study
Source: BMC Musculoskelet Disord. 2021 Dec 20;22:1048. doi: 10.1186/s12891-021-04935-w (PMC8691027; doi:10.1186/s12891-021-04935-w)
Supplement: Supplementary file 2 — Additional file 2. [file 12891_2021_4935_MOESM2_ESM.docx]

Table Sensitivity Analysis Follow-up vs Non-follow-up: These data compare the initial assessment data of each group, by condition, and by whether the participants went onto the follow-up assessments. Independent t-tests with non-equivalent variance.

| Variables with possible impact on the development of hip OA | GTPS continuing group 1  (n=24)  Mean (SD)  Median [Min, Max] | GTPS non-follow-up group  (n=7)  Mean (SD)  Median [Min, Max] | *Independent T test*  *Mean difference (se)*  *95% CI* |  | ASC continuing group  (n=20)  Mean (SD)  Median [Min, Max] | ASC non-follow-up group  (n=3)  Mean (SD)  Median [Min, Max] | *Independent T test*  *Mean difference (se)*  *95% CI* |
| --- | --- | --- | --- | --- | --- | --- | --- |
| Age (Years) | 51.4 (12.2)  51.5 [28, 73] | 60.3 (14.9)  61 [36, 82] | *8.9 (6.16)*  *[-3.18, 20.96]* |  | 51.9 (8.7)  53.0 [37,72] | 48.0 (8.7)  44.0 [42, 58] | *3.9 (5.39)*  *[-7.30, 15.10]* |
| BMI (kg/m^2^) | 27.0 (5.4)  26.3 [18.9, 39.7] | 29.7 (3.3)  28.6 [26.2, 34.3] | *2.7 (2.15)*  *[-5.96, 0.56]* |  | 24.1 (2.6)  24.0 [19.1, 29.7] | 30.4 (2.3)  30.6 [28.1, 32.6] | *-6.31 (1.58)*  *[-6.5, 15.5]* |
| AQoL-4D (0-1) | 0.775 (0.13)  0.765 [0.54, 0.98] | 0.666 (0.14)  0.680 [0.41, 0.89] | *0.109 (0.059)*  *[-0.007, 0.225]* |  | 0.917 (0.12)  0.950 [0.50, 1.00] | 0.897 (0.18)  1.00 [0.69, 1.00] | *0.02 (0.08)*  *[-0.149, 0.189]* |
| mHHS (0-91) | 68.4 (13.6)  72.0 [47, 87] | 60.6 (9.3)  57.00 [49, 77] | *7.8 (5.5)*  *[-0.98, 16.58]* |  | 88.45 (7.89)  91 [61, 91] | 91 (0.00)  91 [91,91] | *-2.55 (4.65)*  *[-12.21, 7.11]* |
| ODI (0-50) | 19.75 (10.75)   1. [4, 42] | 28.57 (11.06)  28.0 [12, 48] | *-8.82 (4.65)*  *[-18.1, 0.43]* |  | 1.6 (3.59)  0.00 [0.00, 14.0] | 0.00 (0.00)  0.00 [0.00, 0.00] | *1.6 (2.11)*  *[-2.80, 6.00]* |
| FCI (0 – 18) | 2.25 (2.03)  2 [0, 8] | 4.14 (3.33)  4.0 [0,9] | *-1.89 (1.01)*  *[-3.96, 0.182]* |  | 1.0 (1.2)  1.0 [0.0, 4.0] | 1.0 (0.00)   1. [1.0,1.0] | *0.0 (0.73)*  *1 [-1.25, 1.25]* |
|  | n=20 | n=7 |  |  | n=19 | n=3 |  |
| Hip abductor force (N/BM^BMavg^) | 45.4 (13.5)  41.7 [18.6, 72.9] | 36.8 (21.5)  29.6 [14.6, 75.9] | *8.7 (6.7)*  *[-8.1, 25.5]* |  | 52.7 (16.5)  53.8 [24.8. 86.2] | 64.6 (22.8)  56.6 [46.8, 90.3] | *-11.9 (10.5)*  *[-38.5, 14.7]* |
| Hip external rotation strength (N/BM^BMavg^) (n=19) | 22.3 (6.6)  22.7 [8.54, 36.5] | 18.4 (7.2)  15.9 [8.2, 30.1] | *3.9 (2.9)*  *[-2.1, 9.9]* |  | 23.7 (7.0)  23.2 [11.4, 36.5] | 28.4 (8.1)  22.4 [8.2, 35.1] | *-4.7 (4.3)*  *[-14.3, 4.9]* |

Differences at initial assessment. The GT participants who were not able to be followed up reported being older, heavier, weaker, having a lower quality of life, more disability, and more co-morbidities. However, none of these features where statistically different from the continuing group. The ASC participants who were not followed up reported being younger, heavier, stronger, very little difference in quality of life, function or co-morbidities. None of these variables were statistically different from the continuing group. Further, we controlled for age and co-morbidities in the analysis.

NB to calculate the strength data – b/c ID 9 is missing the 11 year F/U ER data, I split the group by 11 year clinical data (ClinicalDataat11yeats=1) but added ID 9 to this group for this calculation.
